# A novel uncultured heterotrophic bacterial associate of the cyanobacterium *Moorea producens* JHB

**DOI:** 10.1186/s12866-016-0817-1

**Published:** 2016-08-30

**Authors:** Milo E. Cummings, Debby Barbé, Tiago Ferreira Leao, Anton Korobeynikov, Niclas Engene, Evgenia Glukhov, William H. Gerwick, Lena Gerwick

**Affiliations:** 1 Division of Biological Sciences, University of California San Diego, La Jolla, CA 92093 USA; 2 Center for Marine Biotechnology and Biomedicine, Scripps Institution of Oceanography, University of California San Diego, La Jolla, CA 92093 USA; 3 Department of Statistical Modelling, St. Petersburg State University, Saint Petersburg, Russia; 4 Center for Algorithmic Biotechnology, St. Petersburg State University, Saint Petersburg, Russia; 5 Department of Biological Sciences, Florida International University, Miami, FL 33199 USA; 6 Skaggs School of Pharmacy and Pharmaceutical Sciences, University of California San Diego, La Jolla, CA 92093 USA

## Abstract

**Background:**

Filamentous tropical marine cyanobacteria such as Moorea producens strain JHB possess a rich community of heterotrophic bacteria on their polysaccharide sheaths; however, these bacterial communities have not yet been adequately studied or characterized.

**Results and discussion:**

Through efforts to sequence the genome of this cyanobacterial strain, the 5.99 MB genome of an unknown bacterium emerged from the metagenomic information, named here as Mor1. Analysis of its genome revealed that the bacterium is heterotrophic and belongs to the phylum Acidobacteria, subgroup 22; however, it is only 85 % identical to the nearest cultured representative. Comparative genomics further revealed that Mor1 has a large number of genes involved in transcriptional regulation, is completely devoid of transposases, is not able to synthesize the full complement of proteogenic amino acids and appears to lack genes for nitrate uptake. Mor1 was found to be present in lab cultures of M. producens collected from various locations, but not other cyanobacterial species. Diverse efforts failed to culture the bacterium separately from filaments of M. producens JHB. Additionally, a co-culturing experiment between M. producens JHB possessing Mor1 and cultures of other genera of cyanobacteria indicated that the bacterium was not transferable.

**Conclusion:**

The data presented support a specific relationship between this novel uncultured bacterium and M. producens, however, verification of this proposed relationship cannot be done until the “uncultured” bacterium can be cultured.

## Background

Filamentous cyanobacteria, bathed in seawater and often growing in nutrient-rich environments, are surrounded by diverse communities of heterotrophic bacteria. The heterotrophic bacteria closely associated with cyanobacteria likely consume released nutrients, but may also produce vitamins and other factors useful to cyanobacterial growth, as well as assisting in cycling of CO_2_ and phosphate, or lowering O_2_ levels for oxygen-sensitive processes such as nitrogen fixation [1, 2]. Various studies have classified some of the taxa of heterotrophic bacteria that live in close proximity to cyanobacterial blooms, including common aquatic phyla such as *Proteobacteria, Bacteroidetes, Actinobacteria*, and *Planctomycetes* [3, 4]. Some potentially new species or genera were also located within these samples, which could suggest that some bacteria may have specific relationships with cyanobacteria [3]. However, many of these latter bacteria are also found living independently of cyanobacteria [4], and the makeup of cyanobacterial-associated communities varies based on the location, type of cyanobacteria, and environmental conditions including nutrient availability and temperature [4–6]. The heterotrophic bacterial community around cyanobacterial blooms appears to be directly influenced by the bloom in that the community structure changes over its progression [6]. In fact, if the cyanobacteria are eliminated by a viral infection, the heterotrophic bacterial community drastically shifts [7]. Conversely, heterotrophic bacteria have also been shown to affect the growth of cyanobacteria. Various strains of bacteria found living with *Nodularia spumigena* were co-cultured with the cyanobacterium, and several were found to either increase or decrease the growth of the cyanobacterium compared to axenic cultures [8]. Additional studies of heterotrophic bacteria associated with cyanobacterial blooms have verified that co-cultures can increase or decrease cyanobacterial growth [9]. This is likely due to specific interactions of carbon and nutrient exchange [10]. However, the interactions between cyanobacteria and natural assemblages of heterotrophic bacteria involve a large number and variety of interfaces, and are certainly more complex than a specific symbiosis involving two specific partners. Thus, it becomes clear that gaps exist in our knowledge of the microbial communities surrounding cyanobacteria.

Cyanobacteria have been a rich source of bioactive natural products (secondary metabolites and/or toxins), and their biosynthesis has been studied at the chemical, biochemical and genomic levels [11–14]. There is some evidence that the bacterial communities associated with cyanobacteria may affect these biosynthetic processes in different ways. Heterotrophic bacterial communities surrounding cyanobacteria may not only change cyanobacterial growth characteristics, but also have the potential to break down cyanobacterial toxins [15] or modulate toxin production. For example, toxic *Microcystis* blooms with different heterotrophic bacteria produce altered microcystins of varying toxicity [16].

Additionally, in some cases there are uncertainties about which organism is the true producer of a natural product, or if a collaborative biosynthetic effort is required between the cyanobacterium and a heterotrophic bacterium. Considering the complexity of these metabolites and their assembly pathways, it is highly unlikely that the pathways separately evolved in such divergent organisms as cyanobacteria and heterotrophic bacteria. For example, the lyngbyatoxins, a class of potent skin irritants and tumor promoters isolated from field collections of *M. producens,* show high structural and pharmacological similarity to teleocidin, a metabolite which is produced by *Streptomyces* species [15, 17]. Similarly, an extract from an assemblage of the cyanobacteria *Moorea producens* and *Tolypothrix* sp. yielded the toxin kalkipyrone; this metabolite is closely related to the *Streptomyces* metabolites actinopyrone and iromycin [18, 19]. Another example is given by swinholide A, an actin-binding toxin originally isolated from the sponge *Theonella swinhoei* [20], but subsequently shown to originate from a member of the complex community of heterotrophic bacteria growing within the sponge [20]. However, swinholide A was also isolated from field collections of a marine cyanobacterium along with a glycosylated derivative [21], initially creating some confusion about the true metabolic source of this complex polyketide. However, recent characterization of closely related gene clusters for swinholide-like molecules from a heterotrophic bacterial symbiont of the sponge, *Entotheonella* sp., and several cultured cyanobacteria, reveals a complex evolutionary relationship between these pathways, and suggest a mixture of vertical inheritance and convergent evolutionary processes [22]. As these examples illustrate, there is considerable uncertainty concerning the true biosynthetic source of secondary metabolites isolated from cyanobacteria that possess natural assemblages of heterotrophic bacteria. Overall, the study of these cyanobacterial-associated heterotrophic bacterial communities is important as it relates to the ecology and physiology of these organisms as well as the roles and production of their secondary metabolites.

*Moorea producens* (previously *Lyngbya majuscula*) [23] is a filamentous tropical marine cyanobacterium capable of photosynthesis but unable to fix atmospheric nitrogen. Members of this genus are known to be prolific producers of natural products; around 200 secondary metabolites have been isolated from this organism, and the genomes of various strains contain many polyketide synthase and non-ribosomal peptide synthetase genes [23–25]. *M. producens* has been observed to possess a large community of bacteria on its filaments [23]. However, very little is known about this bacterial community or its inter-relationships and interactions with the cyanobacterial host. One such strain, *M. producens* JHB, a known producer of the natural products hectochlorin [26], the jamaicamides [27], and cryptomaldamide [unpublished], was originally collected from a shallow habitat in Hector’s Bay, Jamaica in 1996. It has been maintained in uni-cyanobacterial culture since this time along with its associated heterotrophic bacterial community. The metagenome of this *M. producens* JHB strain was sequenced and assembled, followed by extensive binning for cyanobacterial versus heterotrophic bacterial DNA. This process yielded a draft genome of the cyanobacterium along with the essentially complete 5.99 MB genome of a *M. producens* JHB-associated bacterium. Analysis of this latter bacterial genome, along with experiments to determine its identity and potential function as an associate of *M. producens* JHB, is the focus of this current report.

## Methods

### Cyanobacterial cultures

*Moorea producens* JHB (GenBank: FJ151521.1) was collected in Hector’s Bay, Jamaica in August 1996 [27]. *M. producens* 3 L (NR116539) was collected in December of 1993 at Las Palmas Beach near the CARMABI Research Station in Curaçao, Netherland Antilles, N12 07.387' W68 58.157' [24]. 3 L *Oscillatoria* (EU244875), identified as *Oscillatoria nigroviridis,* was isolated as a contaminant of the 3 L *M. producens* strain. *M. bouillonii* (FJ041298) was collected in May of 2005 near Pigeon Island in Papua New Guinea, S4 16.063' E152 20.266'. *Leptolyngbya* sp. (ISBN3Nov94-8, KC207938.1) was collected in November of 1994 near Sulawesi, Indonesia. PAP25Jun12-3 was collected in June of 2012 near Portobello, Panama. All of these were established and maintained as uni-cyanobacterial cultures using standard microbiological isolation techniques [27, 28]. The cultures were grown under static conditions at 28 °C under uniform illumination (4.67 μmol photon s^−1^ m^−2^) with a 16 h/8 h light/dark cycle provided by 40 W cool white fluorescent lights. SWBG-11 media contained 35 g/L Instant Ocean (Aquarium Systems Inc.).

### DNA extraction and sequencing

DNA was extracted from the harvested biomass of cultures of *M. producens* JHB, along with its microbiome of heterotrophic bacteria, using the JGI phenol-chloroform protocol [29]. The metagenomic DNA was sequenced using the Illumina HiSeq system, paired end library of 2 × 100 bp. Approximately 12 GB of data were obtained.

### Assembly and other bioinformatics

The metagenomic reads were assembled using SPAdes version 3.0.0 [30]. The contigs were binned by GC content, coverage, tetranucleotide fingerprint, and phylogenetic classification of 107 single copy genes. This binning strategy strongly suggested that the six largest non-cyanobacterial contigs most likely belonged to the same taxon. Using Geneious De Novo Assembler (Geneious®), an isolated reassembly of these six contigs resolved the repeated regions and generated a circular scaffold comprised of a single contig that only lacked part of the 16S-ITS-23S rRNA operon. However, previous PCR experiments (as described below) had already provided a single and complete 16S rRNA sequence. The final circular scaffold, including the complete 16S rRNA gene, was submitted for automatic annotation using RAST [31]. Numbers of copies of this complete 16S rRNA gene were confirmed by comparing the coverage of a single copy gene found only in this genome (*selA*) versus the coverage of the 16S rRNA gene, confirming that a single 16S rRNA gene was present, and most likely, only a single 16S-ITS-23S operon as well. The identified *selA* gene, which is unique to the Mor1 genome, was used for further experiments as a marker for presence or absence of the Mor1 bacterium. In addition, a more detailed annotation of the Mor1 genome was obtained by submitting the genome to the expert reviewed annotation at JGI (Joint Genome Institute) IMG/ER web platform and to antiSMASH for identification/annotation of secondary metabolite biosynthetic gene clusters [32].

The assembly of the *M. producens* JHB genome was performed using a combination of assembly utilizing SPAdes along with a reference assembly to a closed *Moorea* genome (unpublished). This assembly generated a single scaffold of 9.37 Mb with a 43.5 % GC content. The *M. producens* JHB genome was submitted to the same annotation tools as Mor1, and comparative genomics and statistics between Mor1, *M. producens* JHB, and other genomes were developed using Genome Statistics, Search Pathways, COG Homology and the Abundance Profile tools from the JGI (Joint Genome Institute) IMG/ER database.

The relative abundance of *M. producens* JHB and Mor1 in the overall sequenced metagenomic sample was estimated by the percentage of reads recruited to each of these draft genomes compared to the total number of metagenomic reads (approximately 16 Mb). The genomes were normalized by the average genome size of 3.6 Mb (average size of all 3,777 complete bacterial genomes currently available at JGI database). Similarly, the same percentage was calculated for 421 contigs (maximum size of 10,422 bp, total size of 225,489 bp) not assembled into a genome and not belonging to neither JHB or Mor1 (representing other JHB associates), The recruitment of reads was performed by using Bowtie2 mapping with the option end-to-end and disregarding pair end reads to minimize the exclusion of reads from small contigs. Gene calling using Prodigal was performed and the predicted open reading frames (proteins) were submitted to DarkHorse [33] in order to infer phylogenetic classification for these open reading frames.

### 16S rRNA gene location and analysis

The full length16S rRNA sequence was obtained using PCR. DNA was extracted from *M. producens* JHB cultures using the QIAGEN Genomic-tip 20/G kit and following its standard protocol, and PCR was performed using 25 μL volumes, containing 12.5 μL of 2x *Taq* Master Mix, 0.5 μL MgCl_2_ (25 mM), 1.0 μL of each primer (10 μM), 1.0 μL of DNA template, and 9 μL sterile water. The amplification conditions were as follows: initial denaturation at 95 °C for 4 min, followed by 30 cycles of 95 °C for 30 s, 56 °C with 1 Fw + 1451 Rv or 61 °C with 1 Fw + 899 Rv/1151 Rv for 30 s, and 72 °C for 30 s, followed by a final extension step at 72° for 1 min. Primer sequences are shown in Table 1, and were designed based on the sequence of the 16S rRNA gene from *Escherichia coli* strain K-12. The ensuing PCR product was cloned into the pCR 4-TOPO Vector (Invitrogen TOPO TA Cloning Kit) using the standard protocol, followed by sequencing. The full 16S rRNA sequence was analyzed by BLASTn and RDP Classifier [34] to gain more insights into the phylogenetic characteristics of the unknown organism Mor1.

**Table 1:** Primers designed for use in this study

Primer name	Primer sequence	T_m_ in °C
1 Fw	5′ -AAGGAGGTGATCCAGCCGCAGG- 3′	66.0
899 Rv	5′ -TGAGAGGGTGACCGGCCACACT- 3′	67.0
1151 Rv	5′ -AGGCGACGATGGGTAGCCGACC- 3′	68.0
1451 Rv	5′ -CTGGAGAGTTTGATCCTGGCTCAG- 3′	61.0
*selA* Fw 428	5′ -ACTATCGCAAGGCGATCAACAAGA- 3′	58.6
*selA* Rv 1180	5′ -CTAGCTCATCGCTCCTATCAG- 3′	58.3

A phylogenetic tree based on this 16S rRNA gene was created, incorporating 16S rRNA sequences from *Acidobacteria*, *Proteobacteria*, and *Cyanobacteria*. 16S rRNA sequences were obtained from GenBank, then aligned using MUSCLE aligner with 5 iterations, gap open score −1 and word size of 5 bp. The tree was built using Geneious Tree Builder, with the Jukes-Cantor genetic distance model, Neighbor-joining tree build method, 100 bootstraps, and *Anabaena variabilis* ATCC 29413 as the out-group.

### *Culturing attempts of the associated bacterial community from* Moorea producens *JHB*

Efforts to culture Mor1 separately from filaments of *M. producens* JHB used a variety of solid media containing 2 % agar, as listed in Table 2. Intact or cut filaments of *M. producens* JHB were placed onto each media type. For some culturing trials, *M. producens* JHB filaments were freeze-dried and ground up and then added to the media. Additionally, associated bacteria were washed from the surface of *M. producens* JHB filaments using the following protocol: 2 g of biomass was placed into 10 mL of 0.45 M NaCl, 10 mM KCl, 7 mM Na_2_SO_4_, 0.5 mM NaHCO_3_, and 10 mM EDTA. Added to this was 0.1 mL filter-sterilized Rapid Multienzyme Cleaner (3 M). The sample was then incubated for 2 h at room temperature while shaking at 80 rpm. The sample was vortexed and then centrifuged at 300 × g for 15 min. An aliquot of the supernatant (50–100 μL) containing associated bacteria was then plated onto the various types of media.

**Table 2:** Solid media utilized for culturing heterotrophic bacteria associated with *M. producens* JHB sheaths

Media name	Media content	Enrichment
SWBG-11	SWBG-11	N/A
Enriched SWBG-11	SWBG-11	0.4 % glucose
MA	Difco Marine Agar	N/A
SSS	3 % Sigma Sea Salt + 0.4 % mannose + 0.3 % casamino acids	N/A
Enriched SSS	3 % Sigma Sea Salt + 0.4 % mannose + 0.3 % casamino acids	0.5 μM or 2 μM Ferric Ammonium Citrate
A1	1 % starch + 0.2 % yeast extract + 0.4 % peptone	N/A
SWBG-11	SWBG-11	Media mixed with cut up filaments of *Moorea producens* JHB
SWBG-11	SWBG-11	Media mixed with freeze dried and ground up filaments of *Moorea producens* JHB

Bacterial colonies that grew on these plates were isolated and grown overnight in liquid media. DNA was extracted from the overnight cultures using the Wizard Genomic DNA Purification Kit (Promega). PCR was performed on the DNA samples using the *selA* primers (Table 1), and the 16S rRNA 27 F and 1492R primers [34]. PCR was carried out in 25 μL volumes, containing 12.5 μL of 2x *Taq* Master Mix, 0.5 μL MgCl_2_ (25 mM), 1.0 μL of each primer (10 μM), 1.0 μL of DNA template, and 9 μL sterile water. The amplification conditions were as follows: initial denaturation at 95 °C for 4 min, followed by 30 cycles of 95 °C for 30 s, 55 °C for 30 s, and 72 °C for 30 s, followed by a final extension step at 72 °C for 1 min. The 16S rRNA PCR products were then cloned into the pCR 4-TOPO Vector (Invitrogen TOPO TA Cloning Kit) using the standard protocol, followed by sequencing. The obtained sequences were analyzed using BLASTn.

### Electron microscopy

Samples for TEM were prepared using 2 % glutaraldehyde in saltwater (1 h), 2 × 5 min rinses in saltwater, 1 % osmium tetroxide (1 h), 1 × 5 min rinse in 0.15 M cacodylate buffer, 2 × 5 min rinses in ddH_2_0 and 2 % uranyl acetate overnight. This was followed the next day with 2 × 5 min ddH_2_0 rinses. Dehydration was achieved with a graded (20 %, 50 %, 70 %, 90 %, 100 %, 100 %) EtOH series. The samples were then embedded in 50/50 mixture of Spurr’s/EtOH overnight. The next day the samples were incubated in 100 % Spurr’s for 24 h, after which the samples were placed in 100 % fresh Spurr’s for 2 × 1 h and left to polymerize for 48 h. Thin sections (70 nm) were obtained using an Ultracut E microtome (Reichert-Jung, Vienna, Austria) and then placed on 200 mesh fine bar copper grids. The grids were subsequently stained with uranyl acetate and Sato lead. A 1200FX TEM (JEOL, Tokyo, Japan) was used to view the samples.

### Semi-quantitative PCR of DNA from washed and unwashed filaments

One sample of JHB filaments was prepared according to the wash protocol specified above in “Culturing trials of the associated bacterial community of *Moorea producens* JHB”. After centrifugation, the supernatant was removed and the cyanobacterial filaments were used as a “washed filament” sample.

DNA was extracted from the washed filament sample as well as an equivalent mass of unwashed JHB filaments using the Wizard Genomic DNA Purification Kit (Promega) using the standard protocol. PCR was then performed on both samples using the *selA* primers (Table 1). PCR was carried out in 25 μL volumes, containing 12.5 μL of 2x *Taq* Master Mix, 0.5 μL MgCl_2_ (25 mM), 1.0 μL of each primer (10 μM), 1 μL (18.6 ng/μL) of DNA template, and 9 μL sterile water. The amplification conditions were as follows: initial denaturation at 95 °C for 4 min, followed by 28 cycles of 95 °C for 30 s, 55 °C for 30 s, and 72 °C for 30 s, followed by a final extension step at 72° for 1 min. After PCR, the samples were run on a 1 % agarose gel and the intensities of the bands quantified using Gel Quant Express (Life Technologies).

### Examination for the presence of Mor1 in other cultures

In order to examine the extent of Mor1 in other laboratory cultures, a possible indication of cross-contamination between cultures, the following were tested for the presence of the *selA* gene (present in Mor1 but not *M. producens*; see Results): *Moorea producens* JHB, *Moorea producens* 3 L, *Moorea bouillonii*, 3 L *Oscillatoria*, *Scytonema hoffmani* “2846 axenic and xenic,” *Leptolyngbya* sp. (coded ISBN3Nov94-8), and PAP25Jun12-2. In each case, DNA was extracted from several grams of wet biomass using the QIAGEN Genomic-tip 20/G kit applying the standard protocol. PCR was performed on the extracted DNA samples using the *selA* primers (sequences indicated in Table 1). PCR was carried out in 25 μL volumes, containing 12.5 μL of 2x *Taq* Master Mix, 0.5 μL MgCl_2_ (25 mM), 1.0 μL of each primer (10 μM), 1.0 μL of DNA template, and 9 μL sterile water. The amplification conditions were as follows: initial denaturation at 95 °C for 4 min, followed by 30 cycles of 95 °C for 30 s, 55 °C for 30 s, and 72 °C for 30 s, followed by a final extension step at 72° for 1 min.

### Co-culturing of the JHB strain with other cyanobacteria

To determine whether Mor1 could be transferred from *M. producens* JHB to other cyanobacteria, co-culturing experiments were performed. The cyanobacteria chosen for co-culture were *M. producens* 3 L, *Oscillatoria*, *Leptolyngbya* sp. (coded ISBN3Nov94-8), and PAP25Jun12-2. The co-cultures and controls were set up as diagrammed in Table 3. Each co-culture or control was grown in duplicate, in 250 mL of SWBG-11 each. After 2 weeks, the co-cultures were separated under sterile conditions using a dissecting microscope, and grown in SWBG-11 until several grams of wet biomass could be obtained for DNA extraction.

**Table 3:** Matrix of co-culturing experiments involving *M. producens* JHB and various other filamentous tropical marine cyanobacteria

	*M. producens* JHB	3 L *Oscillatoria*	*Leptolyngbya* sp.	PAP25Jun12-2
*M. producens* JHB	Single culture control	Mor1 transfer experiment	Mor1 transfer experiment	Mor1 transfer experiment
3 L *Oscillatoria*	X	Single culture control	Mor1-absent control	Mor1-absent control
*Leptolyngbya* sp.	X	X	Single culture control	Mor1-absent control
PAP25Jun12-2	X	X	X	Single culture control

The entries indicate the intent of each co-culturing combination

DNA extraction was performed for each sample utilizing JGI’s phenol-chloroform protocol [29] or the QIAGEN Genomic-tip 20/G kit using the standard protocol. Each DNA sample was then tested for the presence of the 16S rRNA gene (to indicate sample quality) and the *selA* gene using PCR. Primers 27 F and 781R for 16S rRNA [35], and *selA*Fw 428 and *selA*Rv 1180 were used (Table 1). PCR was carried out in 20 μL volumes, containing 10 μL of 2x *Taq* Master Mix, 0.5 μL MgCl_2_ (25 mM), 1.0 μL of each primer (10 μM), 1.0 μL of DNA template, and 6.5 μL sterile water. The amplification conditions were as follows: initial denaturation at 95 °C for 4 min, followed by 30 cycles of 95 °C for 30 s, 50 °C with 16S rRNA or 55 °C with *selA* for 30 s, and 72 °C for 60 s, followed by a final extension step at 72 °C for 7 min.

### Accession numbers

The genome of the bacterium Mor1 has been deposited in GenBank under the accession number CP011806.

## Results and discussion

### Genome assembly and annotation

The non-axenic uni-culture of the cyanobacterium *M. producens* JHB, originally collected in Hector’s Bay, Jamaica, was sequenced along with its associated heterotrophic bacterial community by Illumina HiSeq sequencing, assembly with SPAdes, binned and reassembled with the Geneious *De Novo* Assembler. In addition to a single scaffold for the cyanobacterial genome (to be reported separately), this process yielded a 5.99 Mb contig from an associated bacterium. Average coverage of this bacterial contig was 33.6 fold and it possessed a 66.8 % GC content, very different from the 43.5 % GC content of the *M. prod*ucens JHB genome. Further analysis revealed that the scaffold was circular and lacked only a fraction of the 16S-ITS-23S operon (partial 16S and 23S rRNA genes were present but the full ITS region was absent) comprising 2820 nucleotides between the 5′ and 3′ ends of the circular scaffold. Continued search of the raw sequencing data was unsuccessful to resolve this region. However, sequence data from PCR amplification of the complete 16S rRNA gene was incorporated into the scaffold (hereafter named the Mor1 chromosome).

The assembled complete genome of Mor1 was submitted for rapid automatic annotation through RAST (http://rast.nmpdr.org/), and using RAST’s SEED Viewer, revealed that the genes for photosynthesis or carbon fixation were lacking, thus indicating that it was heterotrophic. Preliminary comparison of the genomes of *M. producens* JHB and Mor1, again using RAST, revealed several genes present in the bacterium but not in JHB. Of these, the L-seryl-tRNA selenium transferase gene (*selA*) was selected for use as a specific genetic marker of Mor1, useful for examining the presence of this organism in other cyanobacterial strains. This gene encodes for the tRNA incorporation of selenium-containing cysteine residues in proteins and is not common in cyanobacteria [36]. Additional inspection of the sequenced metagenome revealed that the *selA* gene was present only in the Mor1 chromosome and as a single copy. Moreover, BLASTN analysis revealed that none of the 30,622 bacterial genomes in the JGI database contains a single sequence with more than 50 % coverage and 90 % identity to the Mor1 *selA* gene, identifying this gene as an excellent specific genetic marker of this bacterium. Consequently, specific primers were created from the sequence of the *selA* gene and utilized in later experiments as described below.

**Fig. 1:**
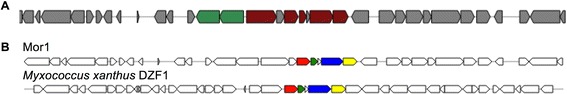
Schematic of the polyketide synthase (PKS) type III pathway discovered within the Mor1 genome using antiSMASH [41]. **a** The entire Mor1 type III PKS gene cluster with maroon genes corresponding to biosynthetic genes, green genes corresponding to regulatory genes, and grey genes corresponding to other uncharacterized genes. This locus is located between nucleotides 1238773 – 1279822 of the genome. **b** The comparison of the Mor1 gene cluster with homologous areas within the alkylresorcylic acid pathway gene cluster in the genome of *Myxococcus xanthus* DZF1. The red arrow corresponds to a stilbene synthase gene with 58 % identity and 99 % coverage; the green arrow corresponds to a methyltransferase gene with 54 % identity and 85 % coverage; the blue arrow corresponds to an AMP-dependent synthetase with 54 % identity and 96 % coverage; the yellow arrow corresponds to a monooxygenase gene with 47 % identity and 69 % coverage. Images generated by antiSMASH [31]

The antiSMASH program was used to identify secondary metabolite pathways within the Mor1 genome [32], and revealed one polyketide type III pathway and one terpene biosynthetic pathway. The polyketide synthase (PKS) type III pathway has high amino acid identity as well as open reading frame organization with the alkylresorcylic acid pathway in *Myxococcus xanthus* [37], as shown in Fig. 1. However, efforts to identify alkylresorcylic acid from the chemical extract of the *M. producens* JHB consortium using the GnPS mass spectral network were not successful [38]. Thus, from gene sequence and MS analyses, Mor1 is not a major producer of recognizable secondary metabolites (e.g. PKS, NRPS or hybrid natural products typical of cyanobacteria).

**Fig. 2:**
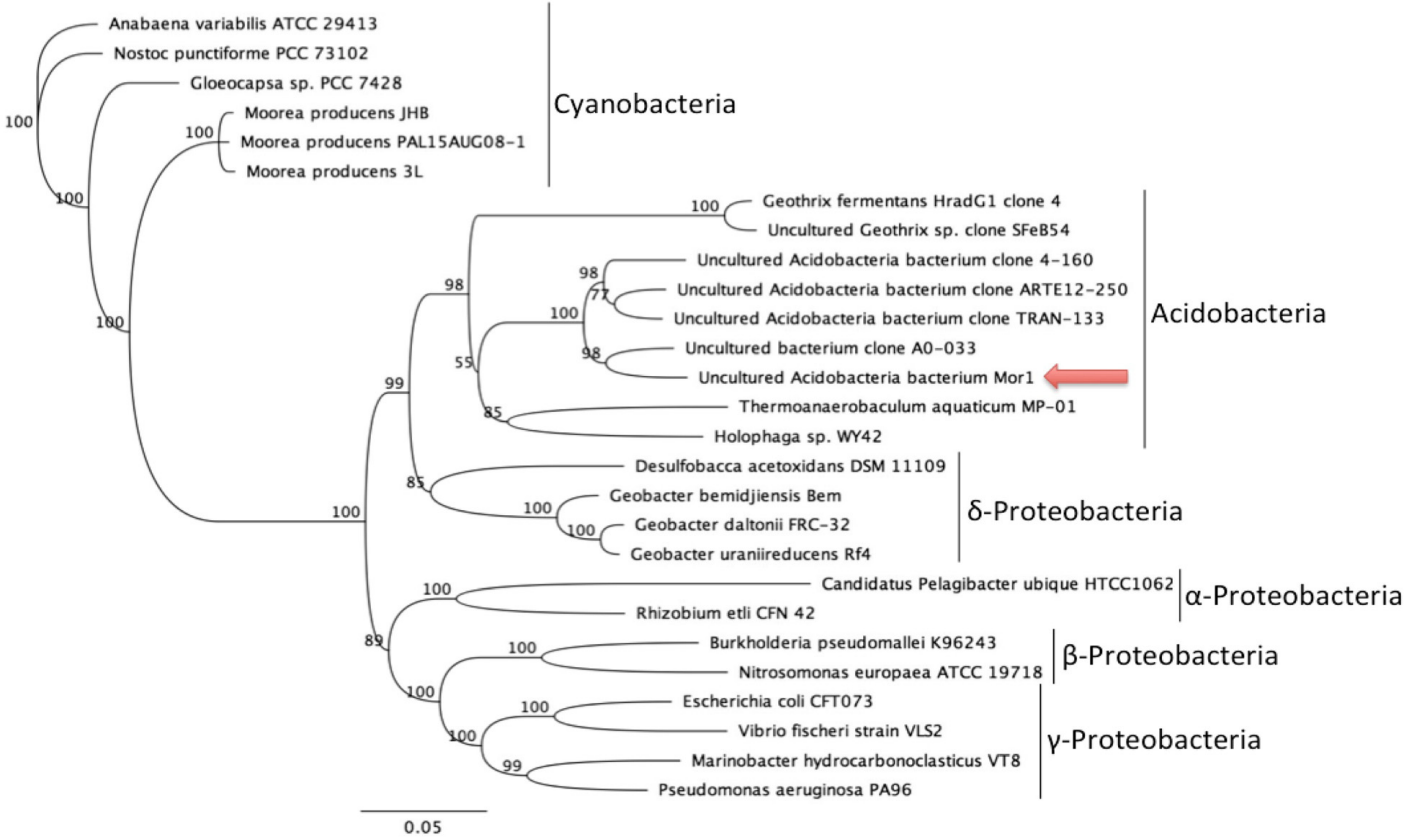
Phylogenetic tree comparing the 16S rRNA sequence of Mor1 to those of other bacteria. *Anabaena variabilis* ATCC 29413 was used as the outgroup. Mor1, indicated by an arrow, clusters with uncultured *Acidobacteria* strains, indicating that it likely belongs to a novel clade of phylum *Acidobacteria*

Comparison of the 16S rRNA sequence of Mor1 to NCBI’s database via BLAST revealed an identity of less then 95 % to an unknown, uncultured bacteria from marine environmental sediment samples. The closest match for a cultured bacterium was *Desulfobacca acetoxidans* (GenBank: NC_015388.1), with an 85 % identity and 100 % coverage. The 16S rRNA sequence was submitted to RDP Classifier for further phylogenetic characterization [34]. This resulted in identification of the organism as belonging to the phylum *Acidobacteria*, subgroup 22 with a 100 % confidence threshold. Further taxonomic classification of *Acidobacteria* subgroup 22 does not currently exist [39, 40]; thus, this bacterium belongs to an, as yet, unnamed and unidentified genus and species. A phylogenetic tree comparing the 16S rRNA sequence of Mor1 with those of other bacteria, including members of *Acidobacteria, Cyanobacteria,* and *Proteobacteria*, is depicted in Fig. 2. Although the phylum *Acidobacteria* is not currently well-classified, members of the phylum have been discovered living within the associated communities of marine sponges and zoanthids, suggesting that marine *Acidobacteria* are capable of complex interactions and symbioses with other organisms [41, 42].

### Relative abundance and estimated consortium composition

**Fig. 3:**
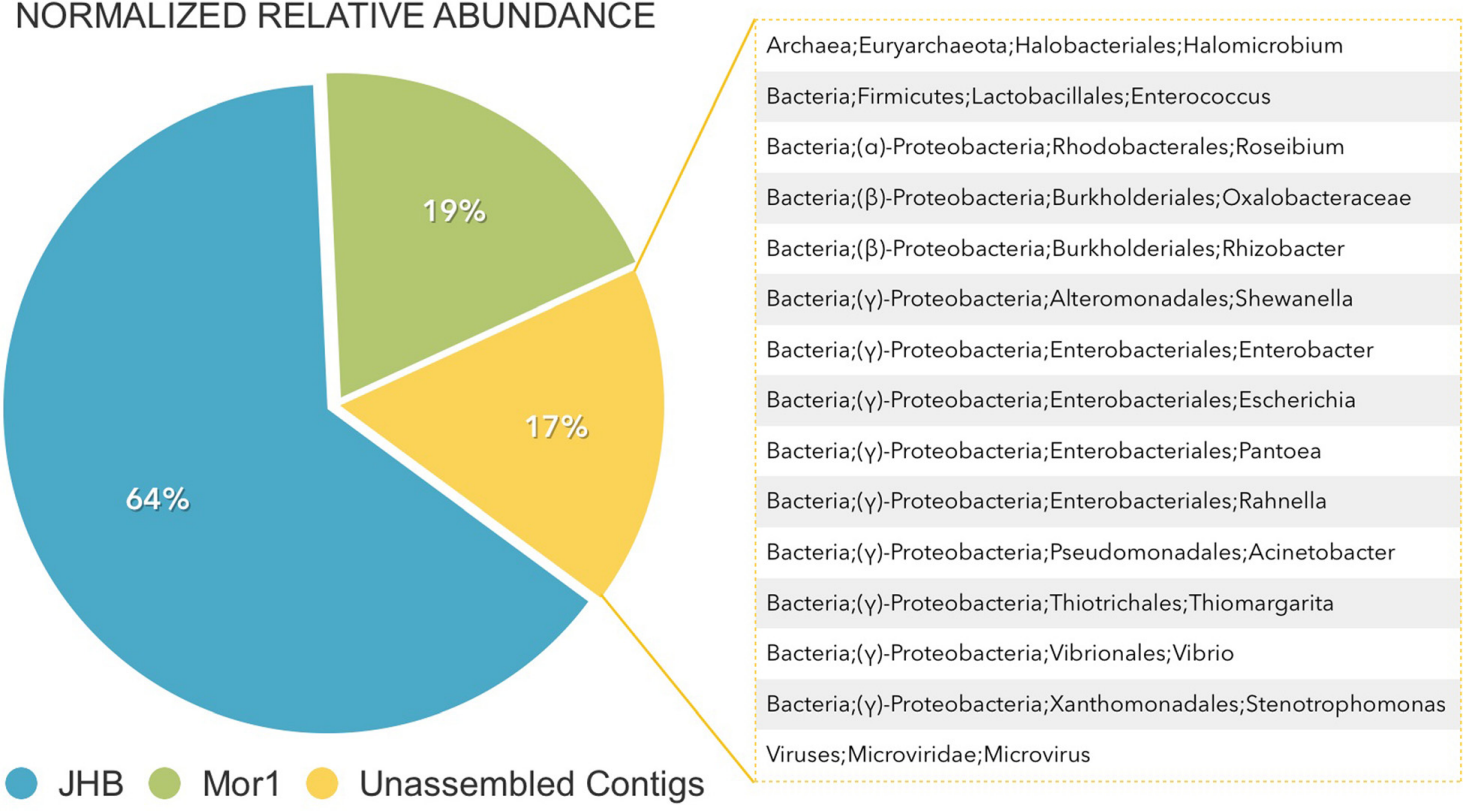
Relative abundance of raw reads belonging to *Moorea producens* JHB, Mor 1 and all the reads unassembled, thus, not belonging to either *Moorea producens* JHB or Mor1

The relative abundance of *M. producens* JHB, Mor1, and other associates was estimated from the recruitment of raw reads. As expected, *M. producens* JHB was the most abundant taxon represented by 64 % of the reads in the metagenomic sample. This was followed by Mor1, with 19 % of the reads. The total relative abundance of all other associates was 17 %, implying that Mor1 is more abundant than the sum of all the other associated heterotrophic bacteria. Phylogenetic classification of these other associates using DarkHorse allowed for a qualitative assessment of the consortium composition (Fig. 3). Unfortunately, the relative abundance of each taxon designated by the DarkHorse analysis cannot be precisely quantified, due to the short lengths of the contigs, which dramatically increase the chances of a highly repetitive gene skewing the read estimation by up to an order of magnitude.

### *Efforts to culture Mor1 free of* M. producens *JHB*

A number of different kinds of media, as described in the Methods and Table 2, were evaluated for Mor1 cultivation. These culture attempts included numerous nutrient combinations as well as enrichments with iron. In order to provide potentially required growth factors for Mor1 culture that might be found in *M. producens* JHB, filaments of JHB were cut into short pieces, freeze-dried and ground with a mortar and pestle, and then added to nutrient agar for culturing experiments. Source bacteria for these culture attempts were obtained from the cyanobacterial sheaths by a wash procedure described in the Methods, and bacteria were cultured from the wash buffer. As a result of these trials, dozens of different bacterial cultures were obtained. By 16S rRNA analysis, these included species of the genera *Muricauda*, *Alteromonas*, *Rhodovulum*, and *Alcinovorax*, as well as *Marinobacter salsuginis* and a *Rhodobacteraceae* strain. However, none of the cultured strains were found to possess the s*elA* gene by PCR analysis, and hence, Mor1 was not among the culturable bacteria from *M. producens* JHB. We propose that Mor1 has nutrient requirements not met by any of these supplemented media types.

### *Mor1 exists mainly on the exterior of the* M. producens *JHB sheath*

**Fig. 4:**
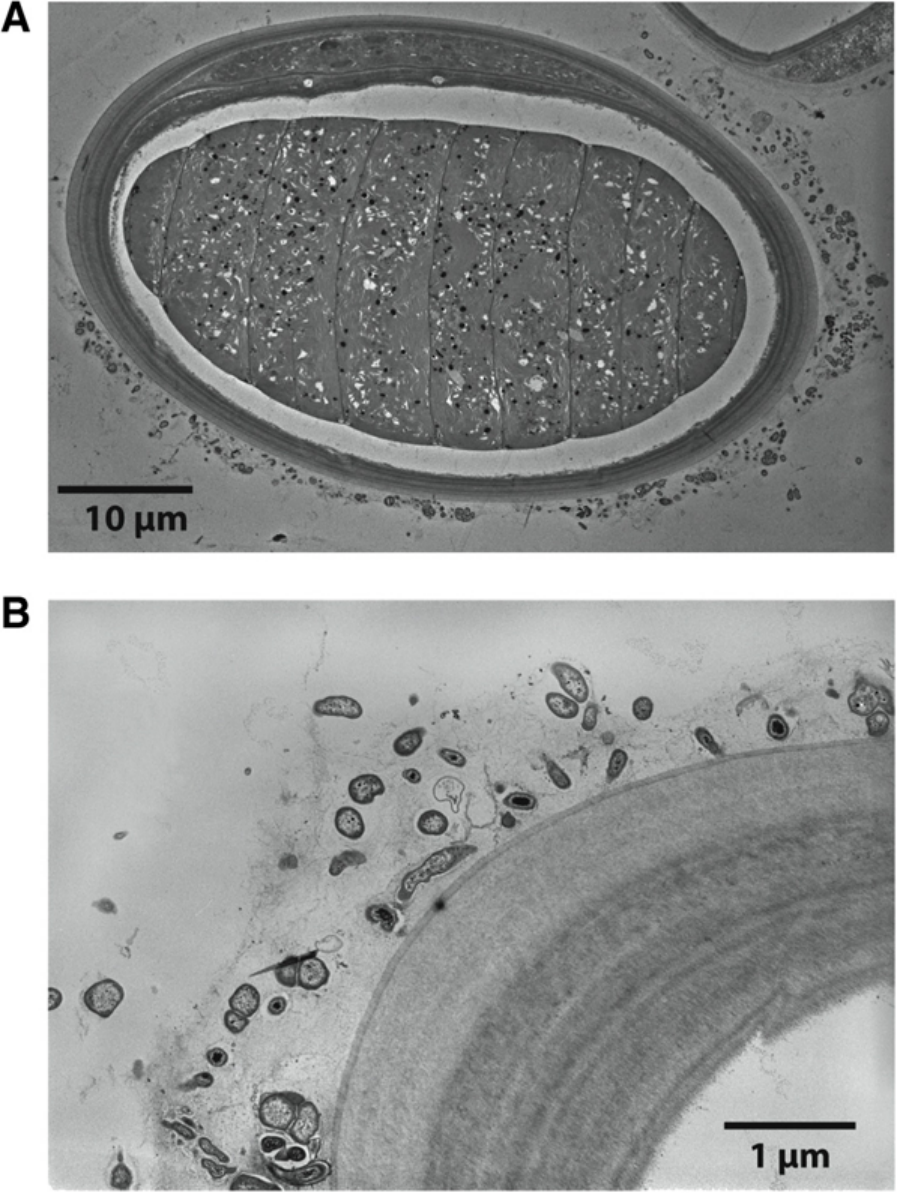
Transmission electron microscopy (TEM) images of 1 *Moorea producens* JHB, showing its polysaccharide sheath and the location of its associated bacterial community. **a** Cross section of a filament of *M. producens* JHB, showing the cell centrally, surrounded by the intermembrane space and polysaccharide sheath. Note that the bacterial growth appears outside the polysaccharide sheath, and not within the intermembrane space. **b** Close-up of bacterial growth on the outside of the polysaccharide sheath

**Fig. 5:**
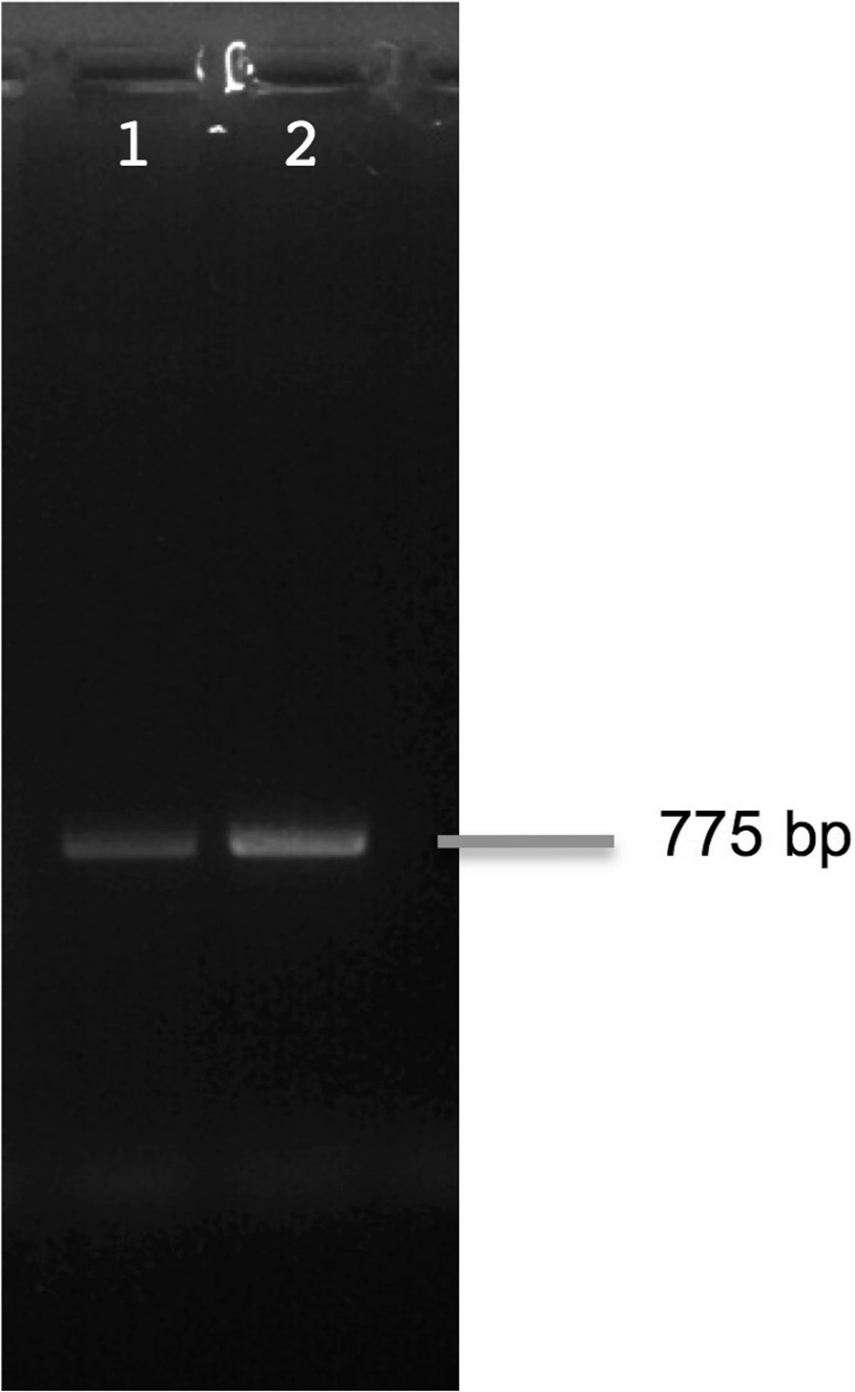
Semi-quantitative PCR of washed and unwashed *M. producens* JHB samples, visualized on a 1 % agar gel. Lane 1: Washed *Moorea producens* JHB sample. Lane 2: Unwashed *Moorea producens* JHB sample. External washing of the filaments reduced the incidence of the Mor1 *selA* gene by an estimated 56.63 % using densitometry

TEM images of cross-sections of JHB filaments are shown in Fig. 4. Bacteria are evident on the outside of the polysaccharide sheath, but the space between the cyanobacterial cell and the sheath appears free of bacteria, and intracellular bacteria are also not in evidence. Thus, Mor1 is likely located on the exterior of the cyanobacterial sheaths. To further explore this hypothesis, we examined two samples of *M. producens* JHB using semi-quantitative PCR of the *selA* gene. One sample contained the intact external bacterial community (unwashed) whereas the second sample was subjected to a wash protocol (described in Methods) designed to remove a substantial fraction of the externally attached bacteria. The *selA* signal was decreased in the washed sample by 56.6 %, indicating that a majority of Mor1 was removed by the washing procedure. Thus, Mor1 exists predominantly on the outside surface of JHB sheaths (Fig. 5).

### *Examination of the specificity of Mor1 on* Moorea spp.

**Fig. 6:**
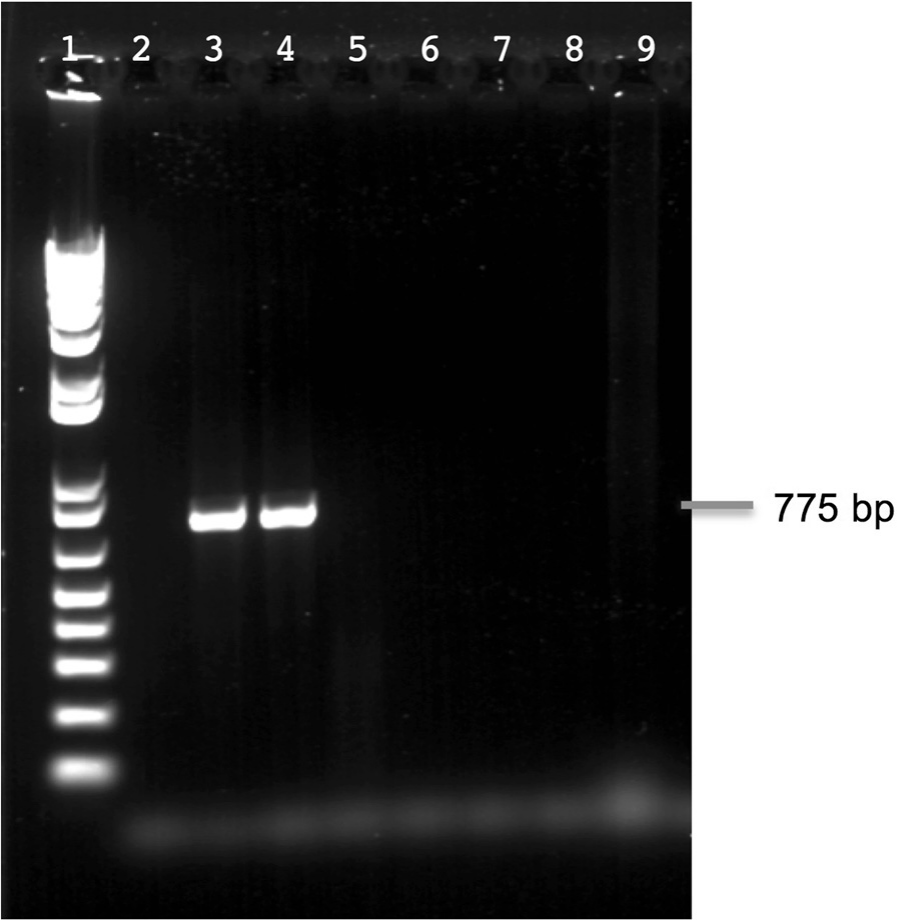
Evaluation of laboratory cyanobacterial cultures for the presence of Mor1. The figure shows the results of PCR with *selA* primers of various laboratory cyanobacterial cultures, run on a 1 % agarose gel. Lane 1: Molecular weight marker (Invitrogen 1 kb Plus DNA Ladder). Lane 2: Negative control, sterile water. Lane 3: *M. producens* JHB. Lane 4: *M. producens* 3 L. Lane 5: *M. bouillonii*. Lane 6: 3 L *Oscillatoria*. Lane 7: PAP25Jun12-2. Lane 8: *Leptolyngbya* sp. Lane 9: *Scytonema hoffmani* “2846 axenic and xenic”

To explore whether Mor1 is a specific associate of *Moorea* and not generally a microbial constituent of our laboratory cyanobacterial cultures, seven different genera/species were tested for the presence of the *selA* gene (see Methods and Fig. 6). The *selA* gene only appeared in cultures of *M. producens* 3 L collected in Curaçao and in *M. producens* JHB from Jamaica, and not in any other of our cyanobacterial cultures, including *Moorea bouillonii* from Papua New Guinea*.* On the basis of this observation, Mor1 was deduced to not be a general laboratory bacterial contaminant in our cultures, and thus we speculated that it is a highly specific associate of *M. producens*.

**Fig. 7:**
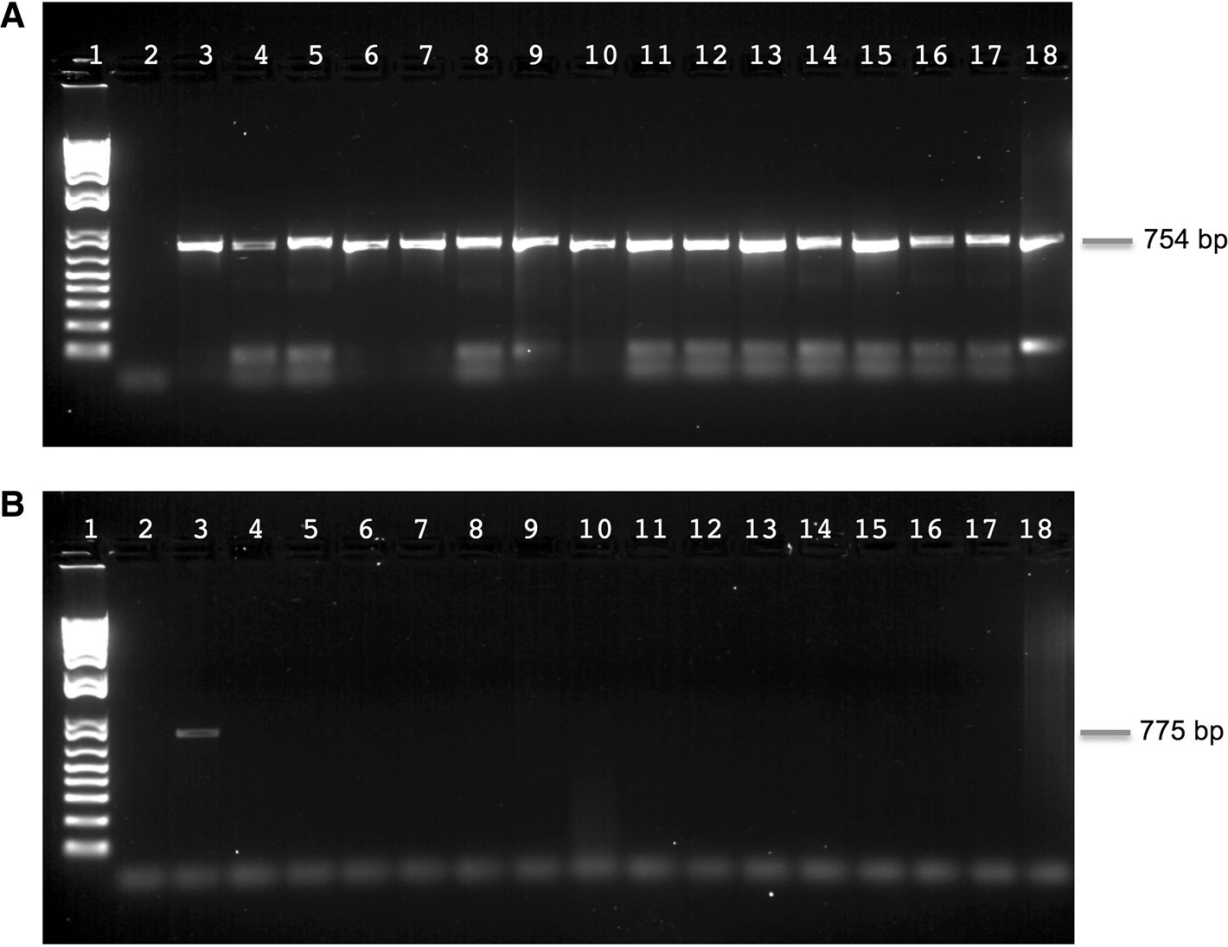
Results of co-culturing M. producens JHB with various other laboratory cultures to evaluate the transferability of Mor1. Shown are 16S rRNA and *selA* PCR reactions using DNA from co-culturing of *M. producens* JHB with other cyanobacteria, visualized on 1 % agar gels. **a** The 16S rRNA PCR of all samples. **b** The *selA* PCR of all samples. Both gels were loaded with the same order of samples for each respective PCR reaction. Lane 1: Molecular weight marker (Invitrogen 1 kb Plus DNA Ladder). Lane 2: Negative control, sterile water. Lane 3: *M. producens* JHB. Lane 4: 3 L *Oscillatoria*. Lane 5: PAP25Jun12-2. Lane 6: *Leptolyngbya* sp. Lanes 7 and 8: 3 L *Oscillatoria* from co-culture with JHB, duplicate co-cultures. Lanes 9 and 10: PAP25Jun12-2 from co-culture with JHB, duplicate co-cultures. 1 Lanes 11 and 12: *Leptolyngbya* sp. from co-culture with JHB, duplicate co-cultures. Lane 13: PAP25Jun12-2 from co-culture with *Leptolyngbya* sp. Lane 14: PAP25Jun12-2 from co-culture with 3 L *Oscillatoria*. Lane 15: *Leptolyngbya* sp. from co-culture with PAP25Jun12-2. Lane 16: *Leptolyngbya* sp. from co-culture with 3 L *Oscillatoria*. Lane 17: 3 L *Oscillatoria* from co-culture with PAP25Jun12-2. Lane18: 3 L *Oscillatoria* from co-culture with *Leptolyngbya* sp

To further explore this hypothesis and to characterize the specificity of the relationship, a set of co-culturing experiments were performed (Table 3). The aim of these was to examine whether Mor1 could be transferred to different genera of cyanobacteria by growing them in co-culture with *M. producens* JHB. Initial PCR screening for the *selA* gene in the “acceptor” species verified that Mor1 was absent and thus exclusive to the *M. producens* JHB culture. *M. producens* JHB was then co-cultured in intimate contact with the strains listed in Table 3 for 2 weeks. The individual strains were then separated and cultured for a variable period to obtain sufficient biomass for DNA extraction and PCR analysis. Two different PCRs were performed on each of the co-culture samples, as shown in Fig. 7. The 16S rRNA gene was used as a positive control that verified that each sample had similar amounts of high-quality DNA (Fig. 7a). Indeed, each sample showed a strong 16S rRNA band of essentially equal intensity. When the same samples were tested for the presence of the *selA* gene, the *selA* gene signal only appeared in the *M. producens* JHB samples and was absent in all of the “acceptor” cyanobacterial cultures that had been co-cultured with JHB (Fig. 7b). From these experiments, we conclude that Mor1 was not transferrable to these other strains, and thus constitutes a specific associate of *M. producens*.

### Genome comparison between M. producens JHB and Mor1

**Fig. 8:**
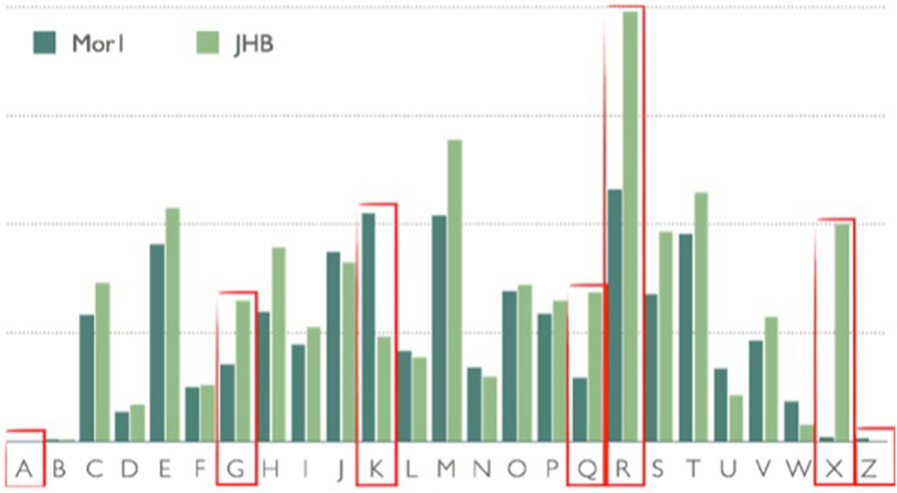
Function category comparison of COGs between Mor1 (dark green) and JHB (light green). Categories that are indicated as different are: A = RNA processing and modification, G = Carbohydrate transport and metabolism, K = Transcription, Q = Secondary metabolites biosynthesis, R = General function prediction only, X = Mobilome (prophages and transposons) and Z = Cytoskeleton

To explore the potential metabolic interactions between *M. producens* JHB and Mor1, the gene abundance profiles of these two organisms were calculated using the online Abundance Profile tool from the IMG/ER website (https://img.jgi.doe.gov/cgi-bin/mer/main.cgi). The two bacteria share 939 clusters of orthologous genes (COGs). The number of COGs exclusive to *M. producens* JHB and exclusive to Mor1 are 549 and 495, respectively. All orthologous genes (OG) are clustered and classified by category in Fig. 8, where substantial differences are highlighted in red between the gene counts and the corresponding cell functions (categories A, G, K, Q, R, X and Z). The categories A and Z represent “RNA processing and modification” and “Cytoskeleton”, respectively, and these categories are highlighted because *M. producens* JHB lacks OG in these categories. Those same genes are missing in other *Moorea* sp. (unpublished) genomes, as well as missing in 90 % of the 345 cyanobacteria from JGI/IMG (larger than 1 Mb) for category Z and around 84 % are missing similar genes in category A. Because these categories of genes appear not to perform essential cell functions in cyanobacteria, they are not considered further in this analysis. Next, categories G, Q and R represent “Carbohydrate transport and metabolism”, “Secondary metabolites biosynthesis, transport and catabolism” and “General function prediction only”, respectively. The number of genes in these categories would be expected to be more numerous in the *M. producens* JHB genome, given it is a larger genome that it also contains many more biosynthetic gene clusters (predicted by antiSMASH to be an astounding 43 biosynthetic gene clusters which account for approximately 22 % of the *M. producens* JHB genome).

Strikingly, category K, “Transcription”, is represented by more than double the OGs in the Mor1 genome versus the JHB genome. This is unexpected as it was previously reported that *M. producens* 3 L (aka *L. majuscula* 3 L) contains a large number of genes involved in transcription and signal transduction. According to this report, “the numbers and diversity of sigma factors that are global regulators of gene expression in bacteria appear higher in *L. majuscula* 3 L than in most other cyanobacteria” [43]. Indeed, *Moorea producens* JHB has the same number of annotated sigma factors as 3 L, 15 in total. Remarkably, the associated Mor1 genome contains 90 sigma-factor genes (the average number of sigma-factor genes among the Acidobacteria genomes currently available at JGI/IMG is 26), 55 of which are annotated as “RNA polymerase sigma-70 factor [a Extra Cytoplasmic Function (ECF) subfamily of factors]. The most important mode of action for ECF sigma in Gram negative bacteria (such as the Acidobacteria) is through Cell Surface Signaling [44]. The other proteins involved in cell surface signaling via the sigma-70 factor are the anti-sigma factor TonB-dependent outer membrane receptor and the TonB-ExbB-ExbD system (see description in reference [43]). The only other receptors found that can be associated with iron metabolism in Mor1 are annotated as “Outer membrane receptor proteins, mostly Fe transport”. *M. producens* JHB lacks this ECF Cell Surface Signaling system, thereby suggesting that the iron acquisition and regulation systems are much more sophisticated in Mor1 than *M. producens* JHB. This hypothesis was explored by adding Ferric Ammonium Citrate to the enriched Sigma Sea Salt media in an attempt to culture Mor1; however, this was unsuccessful.

Lastly, category X [Mobilome (prophages and transposons)] has the most notable difference in gene count between the two bacterial species. The *M. producens* JHB genome possesses 199 transposases whereas none are found in the Mor1 genome. It has been hypothesized that intracellular bacteria have a tendency to accumulate transposases in early stages of intracellular symbiosis [45, 46]. Therefore, the lack of transposases suggests that Mor1 is not an intracellular symbiont; rather, it appears to be extracellular, which is supported by the previously mentioned decrease in signal of the *selA* gene signal when semi-quantitative PCR was performed (Fig. 5). Intriguingly, while analyzing the COGs of 26 other Acidobacteria genomes available at IMG/JGI, it was observed that only one other Acidobacterial genome [JGI GOLD ID: Ga0001215] lacks transposases, indicating that this is an uncommon feature within this phylum. Transposases are important for giving genomes the ability to adapt to evolutionary pressures by facilitating horizontal gene transfer or rearranging of the genome [47]. However, obligate pathogens and endosymbionts have lower numbers of transposases [46]. Hence, the absence of transposases in Mor1 suggests that the potential symbiotic relationship with the *M. producens* JHB strain precludes the need for horizontal gene transfers or rearrangement of the Mor1 genome [48]. Lastly, both *M. producens* JHB and Mor1 harbor a gene from category X known as ParE. This gene is responsible for plasmid stabilization, thus indicating that both organisms may harbor plasmids, even though contigs encoding for plasmids were only found in association with the *M. producens* JHB genome (on the basis of similar GC content). However, it was not possible to completely assemble any plasmids from the metagenomic data due to the fragmented nature of the assembly.

With regards to primary metabolism, Mor1 is only prototrophic for the biosynthesis of L-alanine, L-aspartate, L-glutamate, L-glycine, and L-glutamine, as well as for common co-factors such as flavin, coenzyme A, NAD, heme and thiamine. The lack of biosynthetic genes for a number of key primary metabolites, including several essential amino acids, suggests that Mor1 is adapted to thrive in a consortium with other bacteria, such as with *M. producens* JHB and its microbiome. Specifically, Mor1 lacks biosynthetic genes for several important amino acids: the aromatic amino acids Phe, Tyr and Trp, the positively charged amino acids Lys, Arg and His, and all non-polar amino acids except glycine and alanine. Complementing this, however, is the occurrence in the Mor1 genome of several transporters that are annotated as “amino acid/polyamine/organocation transporter (APC superfamily)”, “amino acid/amide ABC transporter substrate-binding protein (HAAT family)”, and “amino acid transporter”.

Furthermore, it is not capable of cobalamin or biotin biosynthesis, a metabolic insufficiency clearly revealing its dependency on other organisms for survival. Interestingly, a transporter for the uptake of cobalamin was identified in both Mor1 and *M. producens* JHB, which also lacks the capacity for biotin synthesis; this indicates that other bacteria in the consortium are likely providing this key co-factor. Because Mor1 possesses the genes for the biotin carboxyl carrier protein and biotin ligase, biotin is clearly required, but is presumably acquired through uptake from the environment.

**Fig. 9:**
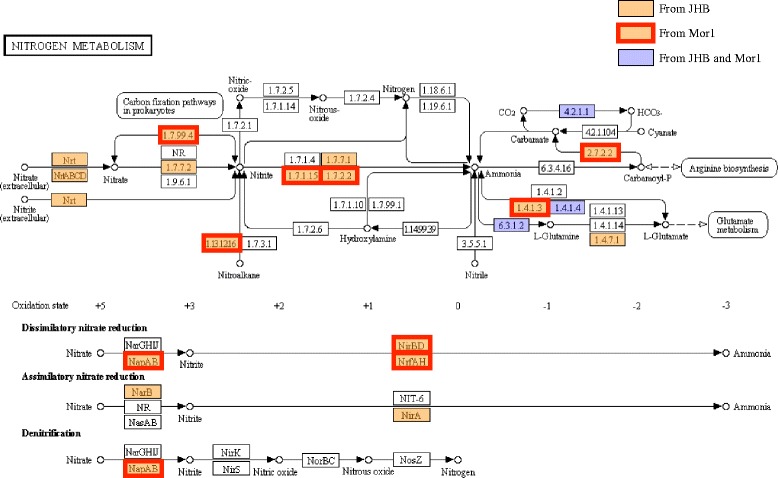
Nitrogen KEGG pathway comparison between **a** *Moorea producens* JHB and **b** Mor1. White boxes represent absent genes, orange represent genes from JHB, Orange box with red line represent genes found in Mor 1 and blue boxes represent homologs of the same gene found in both JHB and Mor1 (http://www.genome.jp/kegg/pathway.html)

In general, the genus *Moorea* is unable to fix nitrogen [43], and by genome analysis of *M. producens* JHB and Mor1, neither of these bacteria possess the required nitrogen fixation genes. However, three interesting Orthologous Groups (OGs) were identified in Mor1 that might be aiding in nitrogen metabolism. The first OG consists of a “uncharacterized protein, possibly involved in nitrogen fixation” (COG3197) whereas the latter two are predicted to be “signal transduction histidine kinases involved in nitrogen fixation and metabolism regulation” (COG5000). Comparison of the nitrogen metabolism KEGG pathway from Mor1 and *M. producens* JHB revealed that they share very few genes (marked in blue, Fig. 9) and that they appear to assimilate nitrogen from very different sources. Whereas *M. producens* JHB possesses the genetic capacity for the uptake of extracellular nitrate and ensuing assimilatory nitrate reduction to produce ammonia and thereby incorporate nitrogen into its amino acids, Mor1 lacks both the nitrate uptake and assimilatory pathways. Rather, Mor1 possesses genes possibly involved in the uptake of ammonium, suggesting that it may rely on acquiring nitrogen from this source as well as by the uptake and recycling of amino acids.

## Conclusions

The sequencing effort of the *M. producens* JHB metagenome revealed a 5.99 Mb genome of an unknown, uncultured heterotrophic bacterium, named here as Mor1. The organism belongs to the phylum *Acidobacteria*, subgroup 22, but is unable to be taxonomically further classified, and as of yet, has not been successfully cultured. A comparative genomics study generated several hypotheses regarding the potential relationship between the *M. producens* JHB and Mor1. Four main areas of interest emerged from this analysis: transcriptional regulation, iron metabolism, nitrogen cycling between the two microbial species, and a complete lack of transposases in the Mor1 genome. Examination for the presence of Mor1 in various laboratory cultures, along with co-culturing experiments to evaluate the transferability of Mor1 to other cyanobacteria, support the idea that it is a specific associate of some strains of *M. producens*.

Because all natural products investigations of *M. producens* JHB to date have occurred using cultured non-axenic biomass which is comprised of cyanobacterial filaments and its associated microbiome, there remains the possibility that some of the identified secondary metabolites (e.g. jamaicamide, hectochlorin) are actually produced by Mor1 or another associated bacterium. Because we have not been able to cultivate Mor1 or the cyanobacterium independent of one another, we are not able to answer this question using chemical methodologies. However, several lines of evidence support the conclusion that these natural products are of cyanobacterial origin: 1) their chemical structures are consistent with pathways known for cyanobacterial natural products, 2) they are produced in relatively high yield per unit of biomass, 3) their biosynthetic pathways use motifs, codons, and GC content consistent with cyanobacterial pathways, and 4) new to this reported work, the major associated heterotrophic bacterium, Mor1, lacks the genes for these metabolic pathways. In fact, only two secondary metabolite pathways were detected in Mor1 based on an antiSMASH analysis of its assembled genome [32], and neither of these is predicted to produce a compound thought to be of cyanobacterial origin.

To further characterize the potential symbiotic relationship between Mor1 and *M. producens*, additional effort is needed to culture *M. producens* and Mor1 independently of one another. Growth rates of *M. producens* with and without Mor1 might infer a symbiotic interaction of these two microbial species [8, 9]. Interaction between these two organisms involving nutrient exchange or signaling molecules could be examined via a transcriptomic analysis [10], imaging mass spectrometry [49], or further chemical analyses. Overall, this work reveals that niche environments such as the sheaths of tropical marine cyanobacteria may be rich locations in which to prospect for novel microbial species with potentially useful biotechnology applications.

## References

[1] Paerl HW (1996). A comparison of cyanobacterial bloom dynamics in freshwater, estuarine and marine environments. Phycologica.

[2] Eiler A, Bertilsson S (2004). Composition of freshwater bacterial communities associated with cyanobacterial blooms in four Swedish lakes. Environ Microbiol.

[3] Hube AE, Heyduck-Söller B, Fischer U (2009). Phylogenetic classification of heterotrophic bacteria associated with filamentous marine cyanobacteria in culture. Syst Appl Microbiol.

[4] Brauer VS, Stomp M, Bouvier T, Fouilland E, Leboulanger C, Confurius-Guns V, Weissing FJ, Stal LJ, Huisman J (2015). Competition and facilitation between the marine nitrogen-fixing cyanobacterium *Cyanothece* and its associated bacterial community. Front Microbiol.

[5] Limei S, Yuanfeng C, Hualin Y, Peng X, Pengfu L, Lingdong K, Fanxiang K (2009). Phylogenetic diversity and specificity of bacteria associated with *Microcystis aeruginosa* and other cyanobacteria. J Environ Sci.

[6] Tuomainen J, Hietanen S, Kuparinen J, Martikainen PJ, Servomaa K (2006). Community structure of the bacteria associated with *nodularia* sp. (cyanobacteria) aggregates in the Baltic Sea. Microb Ecol.

[7] Van Hannen EJ, Zwart G, Van Agterveld MP, Gons HJ, Ebert J, Laanbroek HJ (1998). Changes in bacterial and eukaryotic community structure after mass lysis of filamentous cyanobacteria associated with viruses. Appl Environ Microbiol.

[8] Salomon PS, Janson S, Granéli E (2003). Molecular identification of bacteria associated with filaments of *Nodularia spumigena* and their effect on the cyanobacterial growth. Harmful Algae.

[9] Berg KA, Lyra C, Sivonen K, Paulin L, Suomalainen S, Tuomi P, Rapala J (2009). High diversity of cultivable heterotrophic bacteria in association with cyanobacterial water blooms. ISME J.

[10] Beliaev AS, Romine MF, Serres M, Bernstein HC, Linggi BE, Markillie LM, Isern NG, Chrisler WB, Kucek LA, Hill EA, Pinchuk GE, Bryant DA, Wiley HS, Fredrickson JK, Konopka A (2014). Inference of interactions in cyanobacterial-heterotrophic co-cultures via transcriptome sequencing. ISME J.

[11] Wiegand C, Pflugmacher S (2004). Ecotoxicological effects of selected cyanobacterial secondary metabolites, a short review. Toxicol Appl Pharmacol.

[12] Gerwick WH, Moore BS (2012). Lessons from the past and charting the future of marine natural products drug discovery and chemical biology. Chem Biol.

[13] Jones AC, Gu L, Sorrels CM, Sherman DH, Gerwick WH (2009). New tricks from ancient algae: natural products biosynthesis in marine cyanobacteria. Curr Op Chem Biol.

[14] Jones AC, Monroe EA, Eisman EB, Gerwick L, Sherman DH, Gerwick WH (2010). The unique mechanistic transformations involved in the biosynthesis of modular natural products from marine cyanobacteria. Nat Prod Rep.

[15] Fujiki H, Mori M, Terada M, Sugimura T, Moore RE (1981). Indole alkaloids: dihydroteleocidin B, teleocidin, and lyngbyatoxin A as members of a new class of tumor promoters. Proc Natl Acad Sci.

[16] Dziallas C, Grossart HP (2012). Microbial interactions with the cyanobacterium *Microcystis aeruginosa* and their dependence on temperature. Mar Biol.

[17] Edwards DJ, Gerwick WH (2004). Lyngbyatoxin biosynthesis: sequence of biosynthetic gene cluster identification of a novel aromatic prenyltransferase. J Am Chem Soc.

[18] Graber MA, Gerwick WH (1998). Kalkipyrone, a toxic gamma-pyrone from an assemblage of the marine cyanobacteria *lyngbya majuscula* and *tolypothrix* sp. J Nat Prod.

[19] Surup F, Wagner O, von Frieling J, Schleicher M, Oess S, Muller P, Grond S (2007). The iromycins, a New family of pyridone metabolites from *streptomyces* sp. I. Structure, NOS inhibitory activity, and biosynthesis. J Org Chem.

[20] Bewley CA, Holland ND, Faulkner DJ (1996). Two classes of metabolites from *Theonella swinhoei* are localized in distinct populations of bacterial symbionts. Experientia.

[21] Andrianasolo EH, Gross H, Goeger D, Musafija-Girt M, McPhail K, Leal RM, Mooberry SL, Gerwick WH (2005). Isolation of swinholide A and related glycosylated derivatives from two field collections of marine cyanobacteria. Org Lett.

[22] Ueoka R, Uria AR, Reiter S, Mori T, Karbaum P, Peters EE (2015). Metabolic and evolutionary origin of actin-binding polyketides from diverse organisms. Nat Chem Biol.

[23] Engene N, Rottacker EC, Kastovsky J, Byrum T, Choi H, Ellisman MH, Komarek J, Gerwick WH (2012). *Moorea producens* gen. nov., sp. nov. and *Moorea bouillonii* comb. nov., tropical marine cyanobacteria rich in bioactive secondary metabolites. Int J of Syst Evol Microbiol.

[24] Chang Z, Sitachitta N, Rossi JV, Roberts MA, Flatt PM, Jia J, Sherman DH, Gerwick WH (2004). Biosynthetic pathway and gene cluster analysis of curacin A, an antitubulin natural product from the tropical marine cyanobacterium *Lyngbya majuscula*. J Nat Prod.

[25] Flatt PM, O’Connell SJ, McPhail KL, Zeller G, Willis CL, Sherman DH, Gerwick WH (2006). Characterization of the initial enzymatic steps of barbamide biosynthesis. J Nat Prod.

[26] Ramaswamy AV, Sorrels CM, Gerwick WH (2007). Cloning and biochemical characterization of the hectochlorin biosynthetic gene cluster from the marine cyanobacterium *lyngbya majuscula*. J Nat Prod.

[27] Edwards DJ, Marquez BL, Nogle LM, McPhail K, Goeger DE, Roberts MA, Gerwick WH (2004). Structure and biosynthesis of the jamaicamides, New mixed polyketide-peptide neurotoxins from the marine cyanobacterium *lyngbya majuscula*. Chem Biol.

[28] Sitachitta N, Marquez BL, Williamson RT, Rossi J, Roberts MA, Gerwick WH, Nguyen VA, Willis CL (2000). Biosynthetic pathway and origin of the chlorinated methyl group in barbamide and dechlorobarbamide, molluscicidal agents from the marine cyanobacterium *Lyngbya majuscula*. Tetrahedron.

[29] Feil WS, Feil H, Copeland A (2012). Bacterial genomic DNA isolation using CTAB.

[30] Bankevich A, Nurk S, Antipov D, Gurevich AA, Dvorkin M, Kulikov AS, Lesin VM, Nikolenko SI, Pham S, Prjibelski AD, Pyshkin AV, Sirotkin AV, Vyahhi N, Tesler G, Alekseyev MA, Pevzner PA (2012). SPAdes: a New genome assembly algorithm and its applications to single-cell sequencing. J Comput Biol.

[31] Overbeek R, Olson R, Pusch GD, Olsen GJ, Davis JJ, Disz T, Edwards RA, Gerdes S, Parrello B, Shukla M, Vonstein V, Wattam AR, Xia F, Stevens R (2014). The SEED and the rapid annotation of microbial genomes using subsystems technology (RAST). Nucleic Acids Res.

[32] Blin K, Medema MH, Kazempour D, Fischbach MA, Breitling R, Takano E, Weber T (2013). antiSMASH 2.0 - a versatile platform for genome mining of secondary metabolite producers. Nucleic Acids Res.

[33] Podell S, Gaasterland T (2007). DarkHorse: a method for genome-wide prediction of horizontal gene transfer. Genome Biol.

[34] Wang Q, Garrity GM, Tiedje JM, Cole JR (2007). Naïve Bayesian classifier for rapid assignment of rRNA sequences into the New bacterial taxonomy. Appl Environ Microbiol.

[35] Nubel U, Garcia-Pichel F, Muyzer G (1997). PCR primers to amplify 16S rRNA genes from cyanobacteria. Appl Environ Microbiol.

[36] Romero H, Zhang Y, Gladyshev VN, Salinas G (2005). Evolution of selenium utilization traits. Genome Biol.

[37] Hayashi T, Kitamura Y, Funa N, Ohnishi Y, Horinouchi S (2011). Fatty acyl-AMP ligase involvement in the production of alkylresorcylic acid by a *myxococcus Xanthus* type III polyketide synthase. Chem BioChem.

[38] Wang M, Carver JJ, Phelan VV, Sanchez LM, Garg N, Peng Y (2016). Sharing and community curation of mass spectrometry data with global natural products social molecular networking. Nat Biotechnol.

[39] Barns SM, Cain EC, Sommerville L, Kuske CR (2007). *Acidobacteria* phylum sequences in uranium-contaminated subsurface sediments greatly expand the known diversity within the phylum. Appl Environ Microbiol.

[40] Navarrete AA, Kuramae EE, de Hollander M, Pijl AS, van Veen JA, Tsai SM (2012). Acidobacterial community responses to agricultural management of soybean in Amazon forest soils. FEMS Microbiol Ecol.

[41] Webster NS, Luter HM, Soo RM, Botte ES, Simister RL, Abdo D, Whalan S (2013). Same, same but different: symbiotic bacterial associations in GBR sponges. Front Microbiol.

[42] O’Connor-Sanchez A, Rivera-Dominguez AJ, De los Santos-Briones C, Lopez-Aguiar LK, Pena-Ramirez YJ, Prieto-Davo A (2014). *Acidobacteria* appear to dominate the microbiome of two sympatric Caribbean sponges and one zoanthid. Biol Res.

[43] Jones AC, Monroe EA, Podell S, Hess WR, Klages S, Esquenazi E, Niessen S, Hoover H, Rothmann M, Lasken RS, Yates JR, Reinhardt R, Kube M, Burkart MD, Allen EE, Dorrestein PC, Gerwick WH, Gerwick L (2011). Genomic insights into the physiology and ecology of the marine filamentous cyanobacterium Lyngbya majuscula. Proc Natl Acad Sci U S A.

[44] Llamas MA, Imperi F, Visca P, Lamont IL (2014). Cell-surface signaling in pseudomonas: stress responses, iron transport, and pathogenicity. FEMS Microbiol Rev.

[45] Moran NA, Plague GR (2004). Genomic changes following host restriction in bacteria. Curr Opin Genet Dev.

[46] Lackner G, Moebius N, Partida-Martinez LP, Boland S, Hertweck C (2011). Evolution of an endofungal lifestyle: deductions from the Burkholderia rhizoxinica genome. BMC Genomics.

[47] Aziz RK, Breitbart M, Edwards RA (2010). Transposases are the most abundant, most ubiquitous genes in nature. Nucleic Acids Res.

[48] Stucken K, John U, Cembella A, Murillo AA, Soto-Liebe K, Fuentes-Valdés JJ (2010). The smallest known genomes of multicellular and toxic cyanobacteria: comparison, minimal gene sets for linked traits and the evolutionary implications. PLoS One.

[49] Esquenazi E, Dorrestein PC, Gerwick WH (2009). Probing marine natural product defenses with DESI-imaging mass spectrometry. Proc Natl Acad Sci.

